# Distinct Types of Gut Microbiota Dysbiosis in Hospitalized Gastroenterological Patients Are Disease Non-related and Characterized With the Predominance of Either *Enterobacteriaceae* or *Enterococcus*

**DOI:** 10.3389/fmicb.2020.00120

**Published:** 2020-02-11

**Authors:** Aleksander Mahnic, Martin Breskvar, Saso Dzeroski, Pavel Skok, Spela Pintar, Maja Rupnik

**Affiliations:** ^1^National Laboratory for Health, Environment and Food, Department for Microbiological Research, Maribor, Slovenia; ^2^Department of Knowledge Technologies, Jozef Stefan Institute, Ljubljana, Slovenia; ^3^Jozef Stefan International Postgraduate School, Ljubljana, Slovenia; ^4^Centre of Excellence for Integrated Approaches in Chemistry and Biology of Proteins, Ljubljana, Slovenia; ^5^Faculty of Medicine, University of Maribor, Maribor, Slovenia; ^6^Department of Gastroenterology, University Clinical Centre Maribor, Maribor, Slovenia; ^7^Department of Gastroenterology, University Medical Centre Ljubljana, Ljubljana, Slovenia

**Keywords:** gut, dysbiosis, hospitalization, *Enterobacteriaceae*, *Enterococcus*, fungi, machine learning

## Abstract

Typical disease-associated microbiota changes are widely studied as potential diagnostic or therapeutic targets. Our aim was to analyze a hospitalized cohort including various gastroenterological pathologies in order to fine-map the gut microbiota dysbiosis. Bacterial (V3 V4) and fungal (ITS2) communities were determined in 121 hospitalized gastrointestinal patients from a single ward and compared to 162 healthy controls. Random Forest models implemented in this study indicated that the gut community structure is in most cases not sufficient to differentiate the subjects based on their underlying disease. Instead, hospitalized patients in our study formed three distinct disease non-related clusters (C1, C2, and C3), partially explained by antibiotic use. Majority of the subjects (cluster C1) closely resembled healthy controls, showing only mild signs of community disruption; most significantly decreased in this cluster were *Faecalibacterium* and *Roseburia*. The remaining two clusters (C2 and C3) were characterized by severe signs of dysbiosis; cluster C2 was associated with an increase in *Enterobacteriaceae* and cluster C3 by an increase in *Enterococcus.* According to the cluster affiliation, subjects also showed different degrees of inflammation, most prominent was the positive correlation between levels of C-reactive protein and the abundance of *Enterococcus*.

## Introduction

Multiple different host-specific and environmental factors are known to shape a healthy gut microbiota (Falony et al., 2016; Strati et al., 2016; Zhernakova et al., 2016). Major shifts in microbial community composition, also known as dysbiosis, are associated with a loss of important functions, which can lead to adverse effects on human health. Different types of dysbiosis can be associated with genetic markers, diet, stress, or disease. Typical signs of disrupted microbiota include lower diversity, decrease in anti-inflammatory species such as *Faecalibacterium prausnitzii* and increase in different members of *Enterobacteriaceae* (Belizário and Faintuch, 2018). Some mechanisms leading to state of dysbiosis have already been described (Levy et al., 2017; Rivera-Chávez et al., 2017; Belizário and Faintuch, 2018). However, identification of type or disease-specific microbial signatures in dysbiosis has proven to be challenging.

Microbiota association with gastrointestinal diseases including inflammatory bowel disease (IBD) (Ni et al., 2017; Sun et al., 2019), colorectal cancer (Kelly et al., 2018; De Almeida et al., 2019), and different infections (Lazar et al., 2018) has been extensively investigated. Usually the research is focused on the characterization of the bacterial community; however the remaining members of gut microbiota such as viruses (Kernbauer et al., 2014; Norman et al., 2015), archaea (Chehoud et al., 2015; Lewis et al., 2015), and especially fungi (mycobiota) (Liguori et al., 2016; Sokol et al., 2016) have been lately gaining in recognition.

An important shortfall of the abovementioned studies is the inclusion of a single disease patient cohort. Such an approach might hamper the ability to distinguish between disease-specific microbial patterns and general dysbiosis-associated changes in the gut environment. In an effort to address this issue, we analyzed the bacterial and fungal microbiota in a hospitalized cohort with a variety of gastroenterological pathologies.

Hospitalization by itself introduces numerous alterations to the normal lifestyle irrespective of the underlying disease. Additionally, age, gender, medication, stress, and changed diet potentially influence the microbiota composition, affecting the identification of disease-specific patterns (Falony et al., 2016; Zhernakova et al., 2016). For this reason, we supported conventional analysis of microbial communities with machine learning approaches, allowing us to evaluate the individual and combined ability of different microbiota components at predicting diagnosis-based groups of hospitalized patients, while accounting for the effects of host-specific factors.

## Materials and Methods

### Study Design and Specimen Collection

Stool samples were collected from patients hospitalized at the Department of Gastroenterology at the University Medical Centre Maribor after informed consent. The department has 35 beds and 1,400 hospitalized patients per year. Patients were diagnosed with standard medical procedures including clinical, radiological, endoscopic, and histological criteria and were, for the purpose of further analysis, distributed into five groups based on the diagnosis: IBD [subdivided into Ulcerative colitis (UC, *n* = 25) and Crohn’s disease (CD, *n* = 15)], Tumor (*n* = 22), Infection (*n* = 23), and Other (*n* = 36). All IBD patients were in the state of flare-up during the time of the specimen collection. The most common in the “Tumor” group were patients with pancreatic, gastric or liver cancer. The group “Infection” includes patients with pneumonia, cholangitis, hepatitis, gastritis, or pancreatitis. The group “Other” is a diverse population of patients, mostly diagnosed with cirrhosis or peptic ulcer; included in this group are also patients with unidentified abdominal pain. A total of 121 samples were used in the final analysis. Patients over 80 years of age (*n* = 16) and IBD patients in remission (*n* = 7) were removed. Results for C-reactive protein, total leukocytes, and total neutrophils were collected retrospectively from the medical records and the test result closest to the sample collection date was taken for each patient.

Stool samples from hospitalized patients (HPs) were collected in sterile containers and immediately transported to the laboratory. Samples were homogenized and volumetric equivalent of 50 μl was added to 1 ml of Inhibitex buffer (QIAamp Fast Stool DNA Mini Kit, Qiagen) and stored at −80°C until further use.

The group of healthy volunteers (non-hospitalized controls, NHCs) was used as a control (*n* = 162). Sample collection is described elsewhere (Mahnic and Rupnik, 2018). Out of 197 collected samples, 162 were chosen for the analysis while 35 subjects were removed based on the following criteria: missing information (*n* = 6), age over 80 years (*n* = 2), surgical procedure on gastrointestinal tract in the last 3 months (*n* = 1), hospitalized in the last 3 months (*n* = 6), gastrointestinal infection in the last 3 months (*n* = 13), diagnosed with IBD (*n* = 1), bacterial or fungal community analysis yielded insufficient number of reads (*n* = 6).

Ethics approval was obtained from the National Medical Ethics Committee separately for the study part on hospitalized patients (KME 95/05/15) and the study part on healthy volunteers (No. KME 81/03/16).

### Isolation of the Total Bacterial DNA, Library Preparation, and Amplicon Sequencing

Total bacterial DNA was extracted from each stool sample using the QIAamp Fast Stool DNA Mini Kit (Qiagen, Hilden, Germany) after mechanical disruption (speed 7,000 for 70s) with the SeptiFast Lyse Kit on MagNA Lyser (Roche).

The V3 V4 hypervariable region of the 16S rRNA gene was amplified using broad-range set of primers 341F (5′-CCTACGGGNGGCWGCAG-3′)–805R (5′-GACTACHVGGGTATCTAATCC-3′). Library preparation was carried out according to 16S Metagenomic Sequencing Library Preparation manual (Illumina).

The Internal Transcribed Spacer 2 (ITS2) was amplified using broad-range set of primers ITS86F (5′-GTGAATCATCGAATCTTTGAA-3′)–ITS4R (5′-TCCTCCGCTTATTGATATGC-3′). Library was prepared according to 16S Metagenomic Sequencing Library Preparation manual (Illumina, CA, USA) with the exception of using Q5 High-Fidelity DNA Polymerase (NEB, Massachusetts, USA) instead of KAPA HiFi HotStart ReadyMix (Kapa Biosystems).

Final library quality and quantity were assessed using the High Sensitivity DNA Analysis Kit, BioAnalyzer (Agilent). Sequencing was performed on the Illumina MiSeq platform with Reagent Kit V3 (2 × 300 bp) (Illumina).

### Data Availability

The sequence data supporting the conclusions of this article are available in the form of combined paired end reads (contigs) on the Metagenomics RAST (MG-RAST) database server^1^ under the project access number mgp86691.^2^ Nine samples did not meet the minimum criteria of 1,000,000 bp per sample as required by MG-RAST and are available upon request from authors.

### 16S rRNA Sequence Analysis

The analysis in mothur (v.1.36.1) (Schloss et al., 2009) was performed according to the MiSeq standard operating procedure (SOP) for Illumina paired end reads. The bacterial 16S rRNA reads were processed using the following criteria: (1) reads were not allowed any ambiguous bases and the maximum homopolymer length was set to 8 base pairs (bp); (2) the reads were aligned against the Silva reference alignment (Release 123); (3) chimeras were identified using the UCHIME algorithm; (4) the classification of reads was performed using the RDP training set (version 16) with 0.80 bootstrap threshold value; and (5) sequences were clustered into operational taxonomic units (OTUs) at 97% similarity cut-off. After quality filtering, we obtained an average depth of 37,780 sequences per sample (min 297 sequences, max 87,916 sequences). We removed reads that were represented in the abundance of less than 0.01% and rarefied each sample to 3,000 sequences. A single sample with less than 3,000 sequences was removed from further analysis.

### ITS2 Sequence Analysis

Fungal ITS2 reads were processed using following criteria: (1) the reads were not allowed any ambiguous bases; (2) reads shorter than 205 bp or longer than 502 bp were removed; (3) reads containing homopolymers longer than 12 bp were removed; (4) ITSx software was used for binning in order to remove non-fungal reads (Bengtsson-Palme et al., 2013); (5) the reads were aligned pairwise using the Needleman-Wunsch method (rewards +1 for a match and penalizes with −1 and −2 for a mismatch and gap, respectively); (6) the sequences were clustered into operational taxonomic units (OTUs) at a 98% similarity cut-off; and (7) the classification was inferred using UNITE ITS database (version 6) with 0.80 bootstrap threshold value. After quality filtering, we yielded an average depth of 19,661 sequences per sample (min 504 sequences, max 73,757 sequences). We removed reads that were represented in the abundance of less than 0.01% and rarefied each sample to 1,000 sequences. Samples with less than 1,000 sequences (*n* = 13) were removed from further analysis.

### Statistical Analysis

Alpha diversity (Shannon index), beta diversity (AMOVA with Bray Curtis distances), and population level analysis (LEfSe) (Segata et al., 2011) were performed in mothur (v 1.36.1). Non-Metric Multidimensional Scaling (NMDS) analysis was done in R (version 3.1.3) with the “Vegan” package using Bray Curtis distances as input. Clusters were obtained with the partitioning around medoids (PAM) method (R, version 3.1.3). The optimal number of clusters was determined based on the highest mean Silhouette coefficient. The R script and output for PAM analysis are available in Supplementary Material.

### Machine Learning for Relating Microbiota Composition to Diagnosis-Based Groups

**Figure 1:**
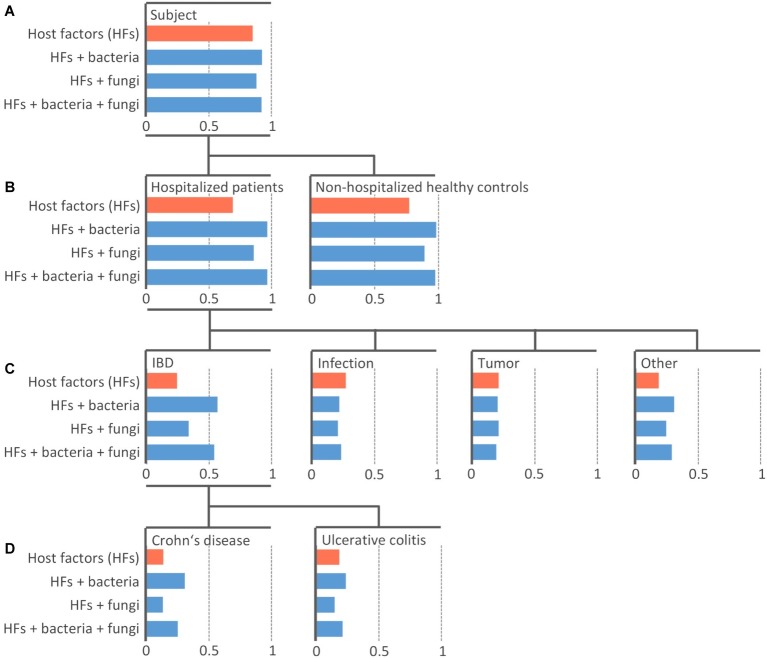
Efficiency of the machine learning models at predicting diagnosis-based groups. Random Forest model of 100 PCTs was constructed individually for four datasets with different sets including host-specific factors alone (orange) or in combination with bacterial and/or fungal community (blue). An individual bar chart is plotted for each diagnosis-based group (node in the tree) arranged into the hierarchy as it was used in the model training. The length of the bar represents the area under the precision-recall curve *AUPRC* The top-most node **(A)** shows the area under the average precision-recall curve $\text{(}AU\bar{PŔC}\text{)}$ scores, indicating the performance of the models across all groups. The other levels **(B–D)** show the *AUPRC* scores indicating the performance of the models for each individual group. The length of blue bars extending to the right of the respective value of model including only host-specific factors (orange bar) represents Δ *AUPRC* – the additional information contained in the microbial community contributing to the prediction of the diagnosis-based group beyond the information provided by host-specific factors.

In this paper, we use machine learning (ML) to relate microbiota composition to diagnosis-based groups. In particular, we built predictive models that take relative OTU abundances as input and predict a specific group as output. Note that the diagnosis-based groups are hierarchically organized (see Figure 1).

The task of predicting a group from such a hierarchy is an example of a hierarchical classification task, where all labels on the path from the root to a leaf of the hierarchy are predicted, rather than just the leaf (e.g., Subject/HP/Tumor, rather than just Tumor). We solve this task by using Predictive Clustering Trees (PCTs), an extension of decision trees that can handle hierarchical classification tasks (Blockeel et al., 1988; Vens et al., 2008). PCTs are grown top-down, from root to the leaves, with each node corresponding to a cluster, increasing within-cluster similarity of the samples (patients) in tree nodes with each consecutive split of the learning data, until only samples (patients) of the same category remain in the leaves, at which point the tree growing stops. PCTs take the diagnosis-based group hierarchy into consideration during the learning process.

In order to lift the predictive performance of PCTs, we learn ensembles thereof by using the Random Forest algorithm (Kocev et al., 2013). This algorithm learns many PCTs, each on a subset of the original data. An ensemble makes predictions by querying each ensemble member (PCT) and averaging their predictions. Genie3 scores were calculated from the ensembles to determine the importance of the individual attributes (OTUs) in the whole dataset (Huynh-Thu et al., 2010).

We evaluated our models in terms of Area Under the Precision-Recall Curve (AUPRC) for individual groups and in terms of Area Under the average Precision-Recall Curve $\text{(}AU\bar{PŔC}\text{)}$ for all groups taken together, i.e., overall model performance (Vens et al., 2008). These measures combine precision and recall and are widely used to determine the quality of machine learning models when dealing with imbalanced learning data.

A more detailed explanation of the used machine learning approaches and evaluation measures used in this article is available in the Supplementary Material.

## Results

Bacterial and fungal stool communities were analyzed in 283 individuals. These included 121 gastroenterological hospitalized patients (HPs) from a single ward and 162 non-hospitalized healthy controls (NHCs). After quality filtering, we obtained a total of 438 bacterial OTUs (102.7 ± 40.6 OTUs per sample), and 136 fungal OTUs (5.8 ± 2.9 OTUs per sample).

Metadata collected on subjects participating in the study included age, gender, and the history of antibiotic therapy in the past 3 months (Table 1). Based on the analysis of the entire studied population, we have shown the correlation between age and antibiotic therapy with the structure of the bacterial community, jointly explaining 1.9% of interindividual variability (PERMANOVA, *p* = 0.004 and 0.002, respectively). Antibiotic therapy was also weakly associated with the fungal community, explaining 1.2% of interindividual variability (PERMANOVA, *p* = 0.013; Supplementary Table S1).

**Table 1:** Distribution of host-specific factors across different groups of hospitalized patients and non-hospitalized healthy controls.

	Gastroenterological hospitalized patients					Non-hospitalized healthy controls
	Crohn’s disease	Ulcerative colitis	Infection	Tumor	Other	
Number of samples	(*n* = 15)	(*n* = 25)	(*n* = 23)	(*n* = 22)	(*n* = 36)	(*n* = 162)
**Age**						
Mean ± SD	41.1 ± 15.0	46.2 ± 14.6	64.6 ± 10.9	69.2 ± 10.9	58.0 ± 14.1	45.1 ± 15.5
**Gender**						
Female [*n* (%)]	9 (60%)	12 (48.0%)	11 (47.8%)	9 (40.9%)	19 (52.8%)	104 (64.2%)
Male [*n* (%)]	6 (40%)	13 (52.0%)	12 (52.2%)	13 (59.1%)	17 (47.2%)	58 (35.8%)
**Antibiotic therapy**						
Yes [*n* (%)]	8 (53.3%)	8 (32.0%)	16 (69.6%)	6 (27.3%)	12 (33.3%)	9 (5.6%)
No [*n* (%)]	7 (46.7%)	17 (68.0%)	7 (30.4%)	16 (72.7%)	24 (66.7%)	153 (94.4%)

### Gastroenterological Hospitalized Patients From Three Distinct Diagnosis Non-related Clusters

Hospitalized patients (HPs) differed significantly from NHCs (AMOVA <0.001). Bacterial community of HPs was characterized by an increase in *Enterobacteriales* and *Lactobacillales* (mainly *Enterococcus*) and reduction in *Clostridiales*, *Bacteroidales*, and community diversity (Supplementary Figure S1). Fungal community of HPs was characterized by an increase in different *Candida* species and reduction in *Saccharomyces cerevisiae* (Supplementary Figure S1). Different diagnosis-based groups of HPs however could not be distinguished among each other neither with bacterial nor fungal community (AMOVA >0.05).

Additionally, significantly higher interindividual variability was observed among diagnosis-based groups of HPs as compared to the NHCs in both bacterial and fungal community (Supplementary Figure S2). In an effort to resolve the unexplained variability, we clustered our samples using partitioning around medoids (PAM). Partitioning into three clusters was determined as optimal based on the highest mean Silhouette coefficient (Supplementary Material).

**Figure 2:**
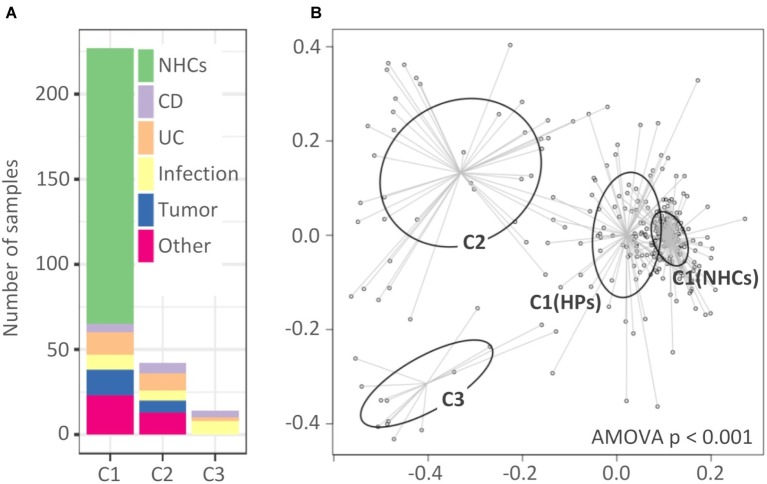
Clusters of hospitalized patients and healthy controls obtained with partitioning around medoids. **(A)** Distribution of samples in clusters based on their diagnosis-based group affiliation. **(B)** Non-metric multidimensional scaling (NMDS) presentation of Bray-Curtis distances. The clusters are highlighted with different colors. Cluster C1 was, for the purpose of downstream analysis, divided into C1(NHCs) and C1(HPs). NHCs, healthy controls; CD, hospitalized patients with Crohn’s disease; UC, hospitalized patients with ulcerative colitis; Other, patients with other diagnoses, primarily liver-associated diseases.

Analysis of molecular variance showed significant differentiation among clusters (AMOVA, *p* < 0.001; Figure 2B). The first cluster (C1, *n* = 227) included all NHCs and 65 HPs, and was for the purposes of downstream analysis split into two sub-clusters, C1(NHCs) and C1(HPs). The second and third clusters (C2 and C3) included 42 and 14 HPs, respectively.

Clusters showed almost no diagnosis association. Clusters C1(HPs) and C2 included individuals from all five target groups of HPs, while C3 included individuals from groups “CD,” “UC,” and “Infection” (Figure 2A). No cluster association was found in relation to age or gender distribution (Supplementary Figure S3). The percentage of subjects on antibiotic therapy differed among clusters [C1(NHCs): 5.6%; C1(HPs): 23.1%; C2: 52.4% and C3: 92.9%, *p* < 0.001] with cluster C3 including only one subject who did not receive antibiotic therapy during hospitalization. However, our results indicate that exposure to antibiotics was not the only driver of cluster-related changes in bacterial and fungal communities (Supplementary Figure S4).

**Figure 3:**
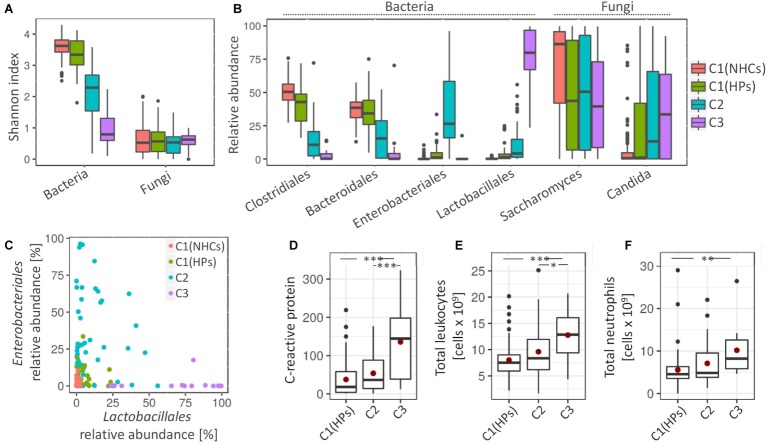
Cluster-specific bacterial and fungal patterns and degree of inflammation. **(A)** Shannon diversity index for bacterial and fungal communities. **(B)** Average relative abundance of four most represented bacterial orders and two most represented fungal genera. **(C)** The relative abundance of *Enterobacteriales* against relative abundance of *Lactobacillales*. Coloring in the subsections **(A–C)** denote cluster affiliation. **(D–F)** Measured inflammation markers, i.e., C-reactive protein (CRP) **(D)**, total number of leukocytes, **(E)** and total number of neutrophils **(F)**. Mean values are highlighted with a red dot. Asterisks denote the significance, at which the distributions differ among the compared groups.

Clusters C2 and C3 showed more pronounced signs of a disrupted bacterial community. Compared to clusters C1(NHCs) and C1(HPs), both C2 and especially C3 showed lower bacterial diversity (Shannon index, *p* < 0.001) (Figure 3A). Cluster C2 was characterized by an increase in different representatives of the family *Enterobacteriales* (*Escherichia/Shigella* (Otu3) and *Enterobacteriaceae* (Otu6); LEfSe, LDA = 5.38 and 5.07, respectively). Detailed investigation of *Enterobacteriaceae* (Otu6) revealed that this OTU includes mainly representatives from genera *Klebsiella* and *Citrobacter.* Cluster C3 was on the other hand characterized by an increase in representatives from the order *Lactobacillales* [*Enterococcus* (Otu4) and *Streptococcus* (Otu19, Otu59); LEfSe, LDA = 5.49, 4.65 and 4.01, respectively] (Figure 3B). Additionally, we have shown that the relative abundances of *Enterobacteriales* and *Lactobacillales* were weakly negatively correlated (Pearson’s *r* = −0.124, *p* = 0.173; Figure 3C).

Cluster C1(HPs) resembled NHCs. Here, less pronounced characteristics of cluster C2 were observed such as decrease in the bacterial community diversity (Shannon index, *p* < 0.001) and an increase in *Enterobacteriales* (Otu3 and Otu6; LEfSe, LDA = 4.14 and 3.72, respectively) and *Streptococcus* (Otu19 and Otu59; LEfSe, LDA = 3.48 and 3.13, respectively). An increase in *Enterococcus* (Otu4), a pivotal characteristic of cluster C3, was on the other hand not observed in cluster C1(HPs). Cluster C1(HPs) was also associated with the reduction in *Clostridiales*, most significantly decreased were *Faecalibacterium* (Otu2) and *Roseburia* (Otu8) (LEfSe, LDA = 4.24 and 4.03, respectively; Supplementary Figure S5).

Regarding fungal community, all three clusters containing HPs showed a decrease in the relative abundance of *Saccharomyces*, predominantly *S. cerevisiae* (Otu1) (LEfSe, LDA > 4.5 for all comparisons). The increase in *Candida*, especially *C. albicans* (Otu2) was significant in clusters C2 and C3 as compared to NHCs (LEfSe, LDA > 4.5 for all comparisons) (Figure 3B).

The three tested inflammatory markers (C-reactive protein (CRP), total leukocytes and neutrophils) were significantly increased in patients belonging to cluster C3 as compared to the cluster C1(HPs), and two of them (CRP and total leukocytes) also compared to cluster C2. No significant differences were observed between clusters C1(HPs) and C2 (Figures 3D–F). *Enterococcus* (Otu4) was significantly correlated with CRP levels independent of the clusters (Pearson’s *r* = 0.463, *p* = 0.002; Supplementary Figure S6), while no community-associated correlations were observed between this OUT and the other two inflammatory markers. No data on inflammatory markers were available for NHCs. Compared to the reference values indicative for the healthy population (CRP: 0.8 mg/L–3 mg/L; total leukocytes: 4–11 × 10^9^ cells/L; neutrophils: 2.5–7.5 × 10^9^ cells/L), the average cluster values suggested an increased CRP in all three clusters containing HPs, while the total leukocytes and neutrophils were elevated above the normal range only in cluster C3.

### Building Random Forest Models to Assess the Ability of Bacterial and Fungal Community to Predict Diagnosis-Based Groups

Predictive Clustering Tree (PCT) models for hierarchical classification (Blockeel et al., 1988; Vens et al., 2008) were trained to identify diagnosis-based groups, which could potentially be predicted by bacterial and fungal microbiota, individually or in combination. In addition to PCTs, where a single decision tree constitutes the predictive model, Random Forest ensembles of 100 PCTs (PCT_RF) were trained (Kocev et al., 2013). This increased the predictive performance over that of single PCT models at the expense of interpretability (Supplementary Table S2).

We built three models with the microbial communities included into the training data along with host-specific factors, i.e., models “Bacteria,” “Fungi,” and “Bacteria + Fungi” (Figure 1). The predictive power of microbiota was assessed by the difference in the AUPRC score between the models learned solely on host-specific factors (Figure 1, orange bars) compared to those including also microbial community data (Figure 1, blue bars). The difference (ΔAUPRC) was interpreted as the information contained in the respective microbial community, which was contributing to the prediction of the diagnosis-based groups beyond the contribution of the available host-specific factors.

### Bacterial Community Is More Informative Than Fungal and Can to a Limited Extent Predict Some Diagnosis-Based Groups

Both bacterial and fungal microbiota showed the ability to distinguish between HPs (PCT_RF, ΔAUPRC = 0.28 and 0.17 for bacterial and fungal community, respectively) and NHCs (PCT_RF, ΔAUPRC = 0.21 and 0.12 for bacterial and fungal community, respectively) (Figure 1B).

Both bacterial and fungal communities were among different diagnosis-based groups most informative at predicting the group “IBD.” Model performance was higher when we trained it on the bacterial community as compared to the fungal community (PCT_RF, ΔAUPRC = 0.32 and 0.09 for bacteria and fungi, respectively). Performance did not improve when we trained it on the combination of both bacterial and fungal community (PCT_RF, ΔAUPRC = 0.30) (Figure 1C).

Bacterial community showed an increased ability to predict the group “CD,” while the ability to predict group “UC” was lower (PCT_RF, ΔAUPRC = 0.17 and 0.05 for CD and UC, respectively). Fungal community showed no ability to predict either group “CD” or “UC” (PCT_RF, ΔAUPRC = 0.0 and −0.04 for CD and UC, respectively) (Figure 1D).

Patients with both CD and UC generally showed similar signs of microbiota disruption, but these were more pronounced compared to other hospitalized patients. For instance, we have shown that although it is indicative of all diagnosis-based groups, the decrease in community diversity, *Faecalibacterium* (Otu2), and *Blautia* (Otu15) was most significant in the group “CD” [LEfSe, LDA = 4.18 and 3.52 for *Faecalibacterium* (Otu2) and *Blautia* (Otu15), respectively; Supplementary Figure S5]. By coupling Random Forest models with the Genie3 score (Huynh-Thu et al., 2010; Petković et al., 2017), we obtained a set of taxa, which were most informative at predicting groups “CD” and “UC” (Supplementary Figure S7); however, significantly larger cohort would be required to validate these results.

Bacterial and fungal communities also showed a limited ability to predict the group “Other” (PCT_RF, ΔAUPRC = 0.12 and 0.06 for bacteria and fungi, respectively) (Figure 1C). This is a heterogeneous group, including (among other) liver-associated diseases, which are known to be associated with changes in the gut microbiota (Sanduzzi Zamparelli et al., 2017; Woodhouse et al., 2018). Neither bacterial nor fungal community showed significant ability to predict patients with infection or tumor (Figure 1C).

## Discussion

Among different gastrointestinal and other chronic diseases, IBD, colorectal cancer, and infection-associated signatures in the gut microbiota have been extensively studied and well described in the past. However, studies commonly compare patient cohorts solely to the healthy controls. Here, we aimed to characterize the gut bacterial and fungal communities in a broader group of gastroenterological patients hospitalized at the same ward.

First we distributed hospitalized patients into diagnosis-based groups. All groups of hospitalized patients differed significantly from healthy controls in both bacterial and fungal community structure. Moreover, our results suggest that most prominent microbial signatures, often reported as disease-specific, are commonly observed in the general population of gastroenterological hospitalized patients. For instance, a decrease in bacterial diversity and multiple members of *Clostridiales* and an increase in *Streptococcus* and *Escherichia/Shigella* were reported in IBD patients as compared to the healthy controls (Gevers et al., 2014; Rajca et al., 2014; Sokol et al., 2016; Takahashi et al., 2016; Halfvarson et al., 2017; Pascal et al., 2017). Also, a significant decrease of *Faecalibacterium* in patients with CD is often described as highly significant (Sokol et al., 2008, 2016; Rajca et al., 2014; Takahashi et al., 2016). In our study, we have shown that these changes are not IBD exclusive, but typically found also in other gastroenterological pathologies. Based on the previously published data, we suggest that these alterations most likely result from the elevated concentration of oxygen in the gut (Rigottier-Gois, 2013).

Random Forest models further confirmed that the ability of the microbial community to predict diagnosis-based groups was limited. By training models on host-specific factors and microbial community data simultaneously, we were able to minimize the effect of age, gender, and antibiotic therapy, factors known to be associated with changes in the gut microbiota (Falony et al., 2016; Zhernakova et al., 2016). Group IBD was the only among diagnosis-based groups where microbial community data improved model performance. However, our sample size was not sufficient to reliably differentiate between general dysbiosis patterns and IBD-specific microbial patterns.

Unsupervised partitioning was subsequently used with the aim to resolve the unexplained variability inside diagnosis-based groups. Three clusters of patients were obtained, which were associated with a different degree of microbiota disruption as well as inflammation observed in patients.

Clusters could not be fully explained either by the underlying disease or the host-specific factors. Antibiotic use correlated with clustering to the greatest extent with the degree of disruption increasing with percentage of patients receiving antibiotics. However, similar patterns of community disruption often occurred in subject receiving antibiotics and those that did not, best exemplified in cluster C2 (Supplementary Figure S4).

The first cluster represented what is generally considered a healthy microbiota. It included all healthy controls and approximately half of the hospitalized cohort. Each of the remaining two clusters represented a unique type of a disrupted microbial community. Cluster C2 was characterized by an increase in the family *Enterobacteriaceae* while the cluster C3 was dominated by order *Lactobacillales*, most prominently genus *Enterococcus.*

Cluster C3 was additionally associated with the most pronounced signs of microbial community disruption such as a lower bacterial diversity, the largest reduction of commensal taxa, and the highest abundance of *C. albicans*. Patients affiliated with this cluster also showed increased values of inflammation markers, most prominently the C-reactive protein and total leukocytes. Additionally, C-reactive protein was strongly correlated with the relative abundance of *Enterococcus*. On the other hand, correlation with an elevated inflammation marker could be secondary to the infection treated with antibiotics, which were a prominent driver of shifts in microbiota.

With available data, we were unable to deduce whether transition from *Enterobacteriales* dominated state (cluster C2) to *Lactobacillales* dominated state (cluster C3) is possible. On one hand, the dysbiosis-associated microbial patterns gradually increase from cluster C1 to C3 with most pronounced signs being observed in the latter. However, the negative correlation between *Enterobacteriales* and *Lactobacillales* (with *Enterococcus* as the main representative) suggests that these could present two independent states associated with different clinical outcomes. Our findings partially explain why the gut microbiota of hospitalized patients is often a suitable reservoir for antibiotic-resistant enterobacteria and enterococci.

All three clusters differed in the prevalence of multiple members of *Clostridiales.* We have shown that some highly prevalent OTUs, for example *Blautia* and *Clostridium*_XIVa, decreased in the abundance exclusively in the instance of severe dysbiosis (clusters C2 and C3). *Faecalibacterium* and *Roseburia* were, on the other hand, significantly decreased also in the group of hospitalized patients resembling healthy controls (cluster C1(HPs)), which suggests they are sensitive to the mild perturbations in the gut microbiota. Both are highly prevalent in the healthy population in Europe and in the USA (Qin et al., 2010; Huse et al., 2012; Falony et al., 2016) and could therefore be considered as potential biomarkers for an early detection of the dysbiosis onset.

Few studies to date have also analyzed the fungal microbiota. The target prediction-associated information contained in the fungal community was in our study limited to the hospitalization-associated increase in *C. albicans* and a decrease in *S. cerevisiae.* In combination with bacteria, fungi showed no additional information contributing to the prediction of diagnosis-based groups. Although studies on fungal microbiota previously associated the increase in *Candida* with IBD (Sokol et al., 2016), its overgrowth seems to be generally associated with the pre-disturbed gut microbiota, for example after the antibiotic treatment (Mavromanolakis et al., 2001).

## Conclusion

We have shown that bacterial and fungal gut microbiota alterations often reported as disease-specific, such as decrease of *Faecalibacterium* and increase of *E. coli*, *Enterococcus,* and *Candida,* are commonly found in a broader population of gastroenterological hospitalized patients. Although gut dysbiosis is often perceived as random (Zaneveld et al., 2017), we have described two distinct types where the severity of disruption was correlated with specific microbial patterns, the degree of inflammation, and to some extent with antibiotic use. We propose *Faecalibacterium* and *Roseburia* as potential biomarkers for an early detection of dysbiosis due to their high sensitivity to mild perturbations in the gut environment and high global prevalence in the healthy population.

## Data Availability Statement

The datasets generated for this study can be found in the MG-RAST https://www.mg-rast.org/mgmain.html?mgpage=project&project=mgp86691.

## Ethics Statement

The studies involving human participants were reviewed and approved by Slovenian National Medical Ethics Committee. The patients/participants provided their written informed consent to participate in this study. Ethics approval was obtained from the National Medical Ethics Committee separately for the study part on hospitalized patients (KME 95/05/15) and the study part on healthy volunteers (No. KME 81/03/16).

## Author Contributions

MR contributed to study concept and design. PS and SP contributed to acquisition of specimen and patient data. AM and SP contributed to DNA extraction and sequencing. AM, MB, and SD contributed to data analysis, machine learning model building and interpretation. AM and MR contributed to drafting of the manuscript. MB and SD contributed to critical revision of the manuscript. All authors have read and approved the manuscript.

### Conflict of Interest

The authors declare that the research was conducted in the absence of any commercial or financial relationships that could be construed as a potential conflict of interest.

## Footnotes

^1^http://metagenomics.anl.gov/

^2^https://www.mg-rast.org/mgmain.html?mgpage=project&project=mgp86691
