# Supplementary material for: Distinct Types of Gut Microbiota Dysbiosis in Hospitalized Gastroenterological Patients Are Disease Non-related and Characterized With the Predominance of Either Enterobacteriaceae or Enterococcus
Source: Front Microbiol. 2020 Feb 11;11:120. doi: 10.3389/fmicb.2020.00120 (PMC7026674; doi:10.3389/fmicb.2020.00120)
Supplement: Supplementary file 1 [file Data_Sheet_1.docx]

**Table S1** Permutational analysis of variance results (PERMANOVA) (R, Vegan package, 1000 permutations) for the bacterial and fungal communities. Statistically significant host covariates are highlighted in bold with asterisks denoting the level of significance.

| **Bacterial community** |  |  |  |  |  |  |  |
| --- | --- | --- | --- | --- | --- | --- | --- |
|  | Df | SumsOfSqs | MeanSqs | F.Model | R2 | Pr(>F) |  |
| Age | 1 | 0.628 | 0.62757 | 2.1884 | 0.00767 | **0.004** | ****** |
| Gender | 1 | 0.233 | 0.23257 | 0.811 | 0.00284 | 0.72128 |  |
| Antibiotic | 1 | 0.973 | 0.97286 | 3.3924 | 0.01189 | **0.002** | ****** |
| Residuals | 279 | 80.01 | 0.28677 |  | 0.9776 |  |  |
| Total | 282 | 81.843 |  |  | 1 |  |  |
|  |  |  |  |  |  |  |  |
| **Fungal community** |  |  |  |  |  |  |  |
|  | Df | SumsOfSqs | MeanSqs | F.Model | R2 | Pr(>F) |  |
| Age | 1 | 0.222 | 0.22171 | 1.0112 | 0.00356 | 0.36763 |  |
| Gender | 1 | 0.152 | 0.15234 | 0.6948 | 0.00244 | 0.58342 |  |
| Antibiotic | 1 | 0.786 | 0.78568 | 3.5833 | 0.0126 | **0.01299** | ***** |
| Residuals | 279 | 61.175 | 0.21926 |  | 0.98139 |  |  |
| Total | 282 | 62.334 |  |  | 1 |  |  |

**Table S2** Model performances in terms of $AUPRC$ scores. The table shows the $AU\bar{PRC}$ scores (pooled for all target groups) in the first row and the $AUPRC$ scores for each target group (label) in the hierarchy individually (Figure 3). Scores are presented for both machine learning approaches, i.e. single Predictive Clustering Trees (PCTs) and Random Forest ensembles of 100 PCTs. Each table section is further divided according to which attributes were included in the training data. Metadata columns (M) concern models where only host specific factors (age, gender, antibiotic therapy) were included. Bacteria (B) and/or Fungi (F) columns specify results for models where microbiota data about the respective population was included in the form of OTU relative abundances.

|  | **Predictive clustering trees (PCTs)** | | | | **Random Forests of PCTs** | | | |
| --- | --- | --- | --- | --- | --- | --- | --- | --- |
|  | M | M+B | M+F | M+B+F | M | M+B | M+F | M+B+F |
| Subjects ($AU\bar{PRC}$) | 0.830 | 0.859 | 0.826 | 0.855 | 0.850 | 0.926 | 0.882 | 0.922 |
|  |  |  |  |  |  |  |  |  |
| Hospitalized  patients (HP) | 0.602 | 0.825 | 0.626 | 0.838 | 0.690 | 0.967 | 0.856 | 0.964 |
| Healthy  controls (HC) | 0.695 | 0.878 | 0.764 | 0.872 | 0.769 | 0.982 | 0.891 | 0.977 |
| ∙ Infection | 0.222 | 0.157 | 0.164 | 0.163 | 0.267 | 0.216 | 0.204 | 0.230 |
| ∙ Other | 0.133 | 0.238 | 0.144 | 0.231 | 0.182 | 0.305 | 0.243 | 0.286 |
| ∙ Tumor | 0.152 | 0.098 | 0.168 | 0.093 | 0.207 | 0.200 | 0.210 | 0.190 |
| ∙ IBD | 0.182 | 0.320 | 0.275 | 0.309 | 0.244 | 0.566 | 0.335 | 0.539 |
| ∙∙ Crohn's disease (CD) | 0.093 | 0.099 | 0.083 | 0.116 | 0.133 | 0.305 | 0.130 | 0.251 |
| ∙∙ Ulcerative colitis (UC) | 0.079 | 0.187 | 0.124 | 0.184 | 0.183 | 0.236 | 0.145 | 0.208 |

**
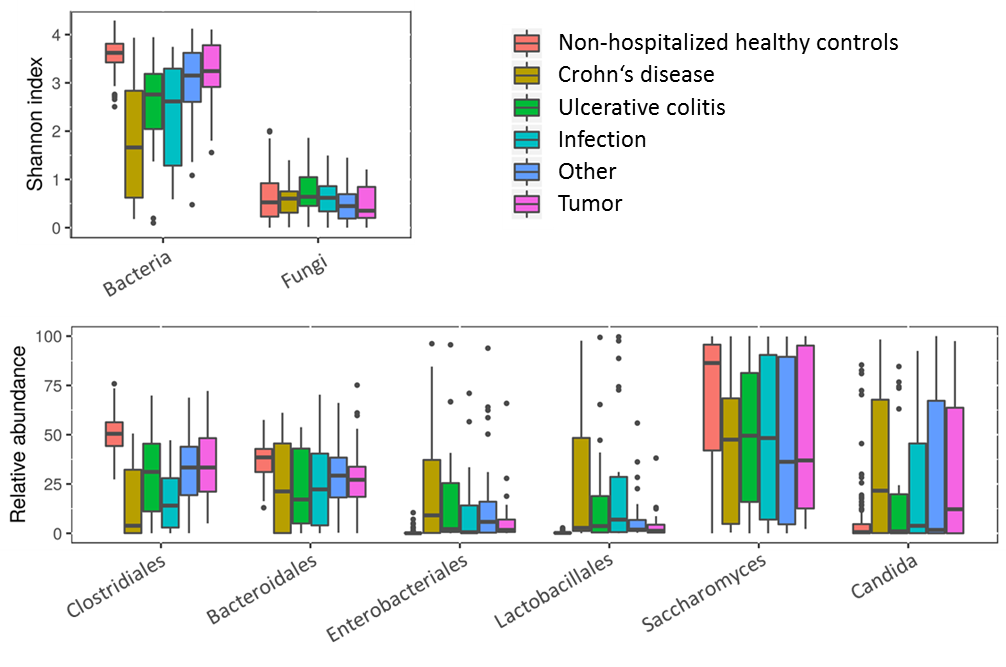
**

**FIGURE S1:** Bacterial and fungal community diversity (Shannon index, top) and relative abundance of 4 most abundant bacterial orders and two most abundant fungal genera (bottom). Colors indicate the diagnosis-based groups, showing that most prominent changes in bacterial and fungal community are comparable among different groups of hospitalized patients as compared to non-hospitalized healthy controls.


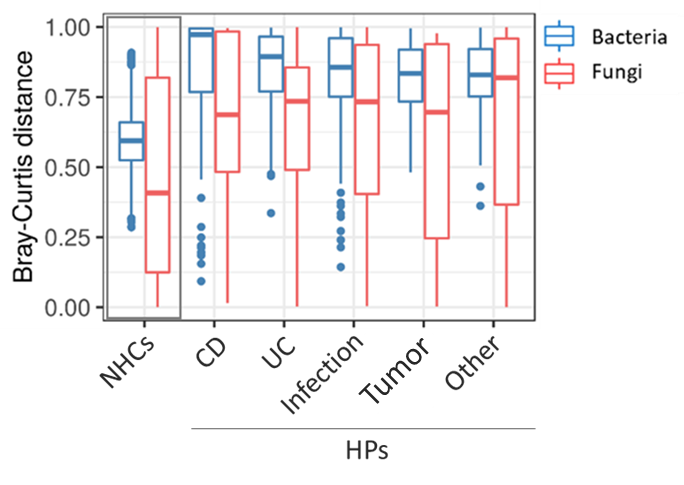


**FIGURE S2:** Internal variability in bacterial and fungal community across target groups. Variability was measured using Bray-Curtis distance. Distributions are presented separately for bacterial (blue) and fungal (red) communities.


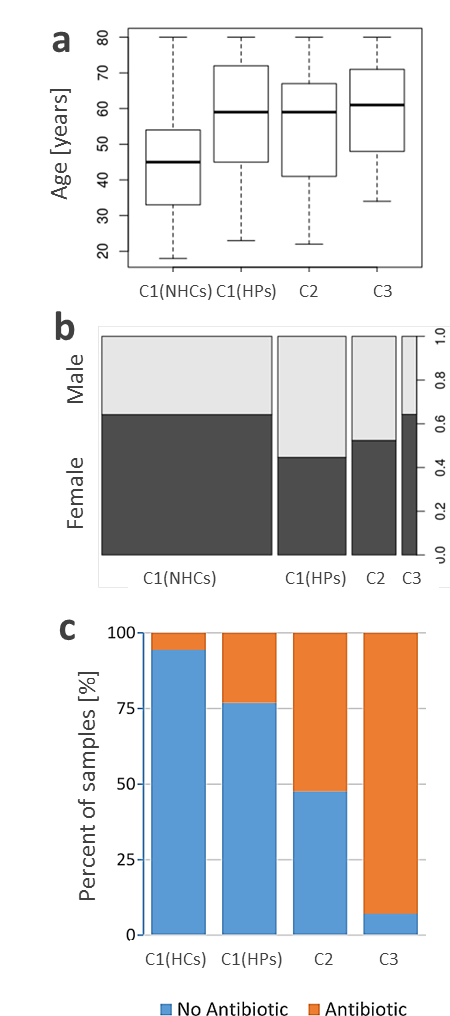


**FIGURE S3:** Age distribution (**a**), gender distribution (**b**) and the proportion of subjects on antibiotic therapy (**c**) according to the cluster affiliation.


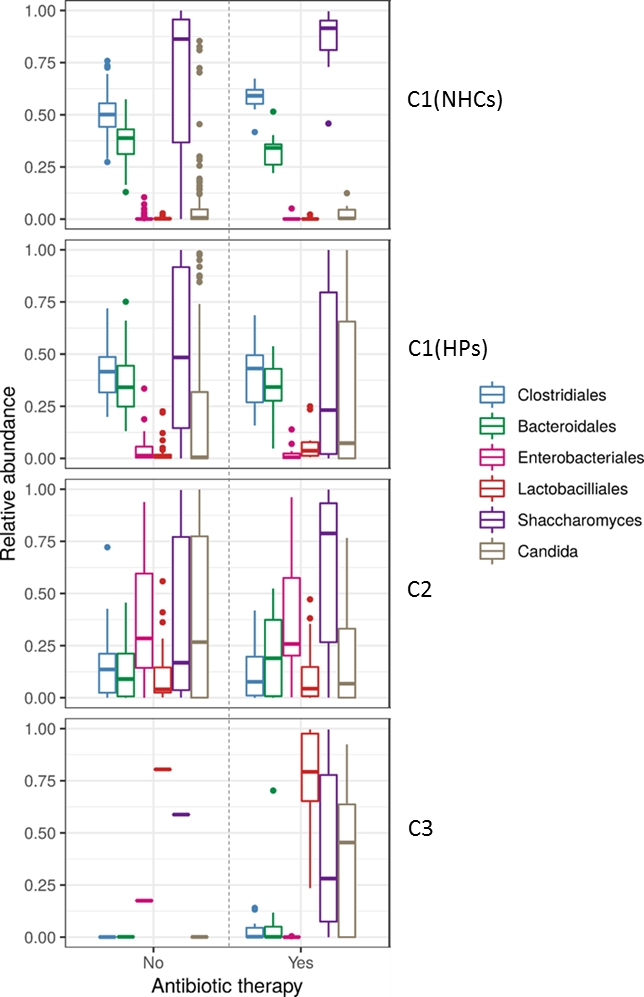


**FIGURE S4:** Bacterial and fungal community according to the exposure to antibiotics shown for each cluster separately. Bacterial community is presented with the four most represented orders while fungal community by two most represented genera. Distribution of each taxa is shown separately for subjects who were not exposed to antibiotics (left) and those who were (right) for each cluster separately (4 levels from top to bottom). Figure shows that the antibiotic associated differences in the community structure are minimal across clusters. This is indicative especially for clusters C1(HPs) and C2 where the percentage of subjects on antibiotic therapy was 23 % and 52 %, respectively. In the cluster C3 all but one subject received antibiotic therapy in the last 3 months which makes the comparison problematic.


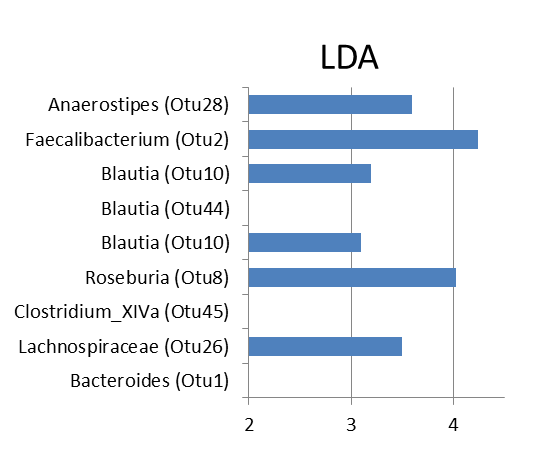


**FIGURE S5:** Comparison of core OTUs between clusters C1(NHCs) and C1(HPs). Bars represent LDA values (LEfSe). Highest differentiation between compared clusters show *Faecalibacterium* (Otu2) and *Roseburia* (Otu8), inidcating that these two core taxa are most sensitive to mild perturbations in gut microbiota.


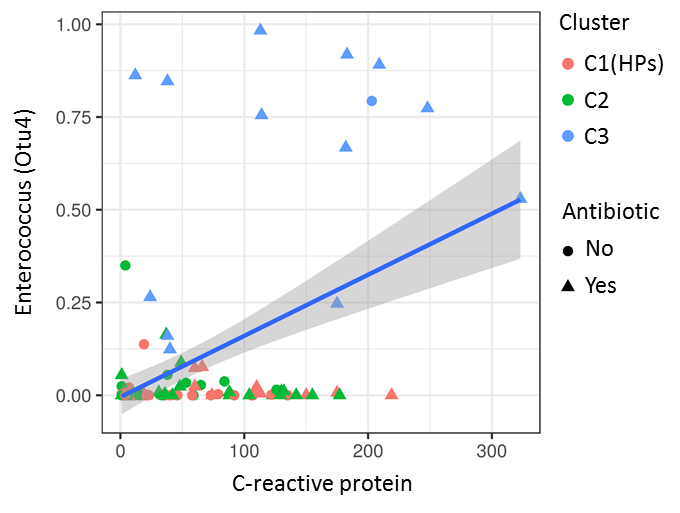


**FIGURE S6:** Correlation between C-reactive protein levels and *Enterococcus* (Otu4) relative abundance. Linear regression is presented with blue line including 95 % confidence interval. Data points are colored according to cluster affiliation and shaped according to antibiotic use.


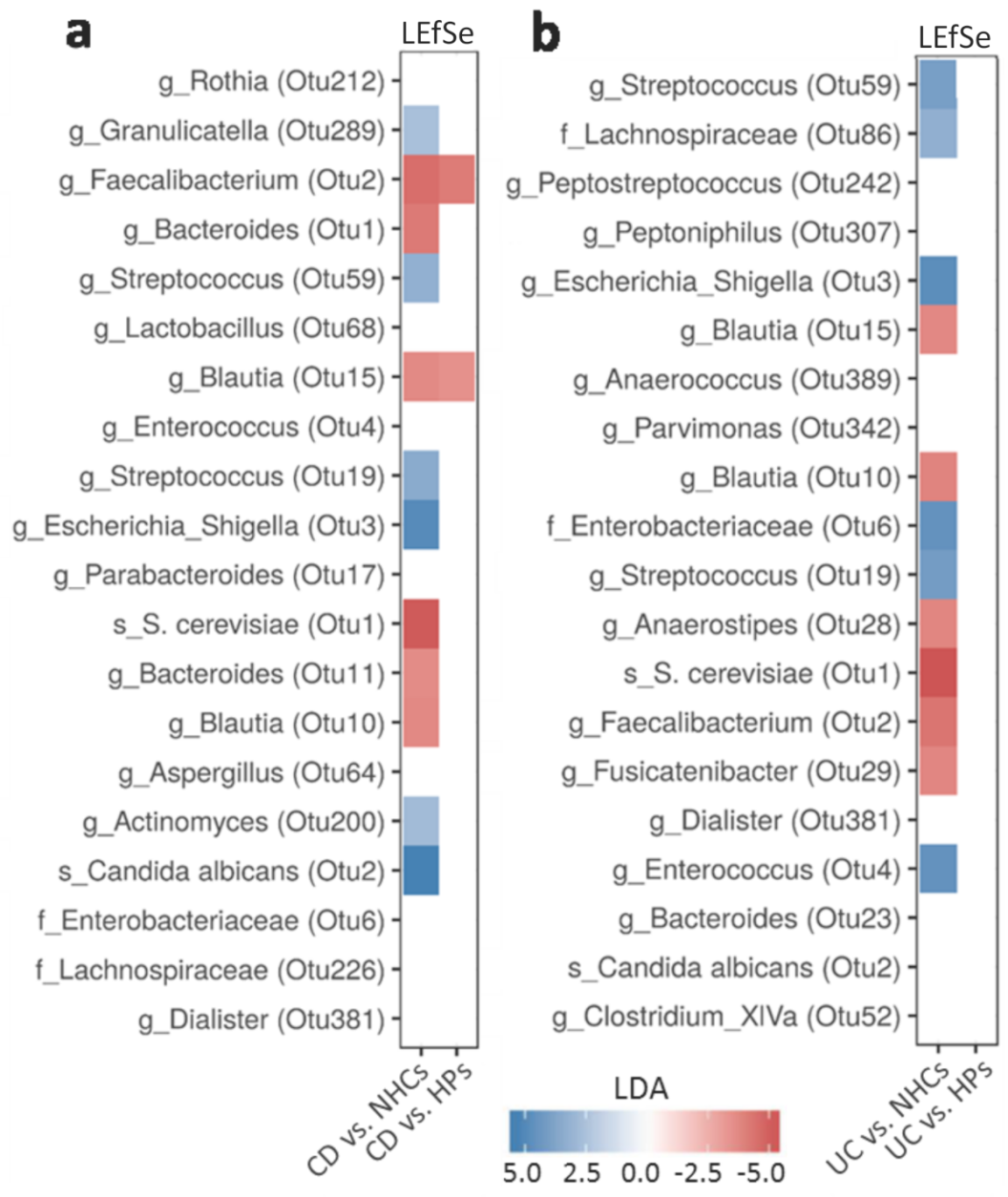


**FIGURE S7. Comparison of bacterial and fungal OTUs predicting the Crohn‘s disease and ulcerative colitis identified by machine learning and LEfSe analysis.** Highest ranked bacterial and fungal OTUs based on their ability to predict hospitalized patients with Crohn‘s disease (a) and ulcerative colitis (b) as obtained by Genie3 score are listed on the left. Heat plots to the right present LDA values obtained with LEfSe test after comparing either CD (**a**) or UC (**b**) group to the healthy controls (NHCs) or the remaining hospitalized patients (HPs). Blue colour indicates the association with either CD or UC, while the red colour indicates the association with either NHCs or remaining HPs. The prefixes in the OUT taxonomic affiliation denote taxonomic level: s, species; g, genus; f, family.
