# Supplementary material for: Distinct Types of Gut Microbiota Dysbiosis in Hospitalized Gastroenterological Patients Are Disease Non-related and Characterized With the Predominance of Either Enterobacteriaceae or Enterococcus
Source: Front Microbiol. 2020 Feb 11;11:120. doi: 10.3389/fmicb.2020.00120 (PMC7026674; doi:10.3389/fmicb.2020.00120)
Supplement: Supplementary file 2 [file Data_Sheet_2.docx]

**R script for PAM and the output:**

library(cluster)

library(vegan)

# Initial data input is the OTU contingency table

data <- read.csv('bacteria.otus.csv', header = T, row.names = 1)

# Create matrix with Bray-Curtis distances.

d.dist <- vegdist(data, method = 'bray')

# For loop will test the supervised clustering with the Partitioning around medoids (PAM)

#Procedure. Optimal clustering will be dicedid upon highest mean Silhouette coefficient

for (i in 2:10){

# Partitioning around medoids

pam <- pam(d.dist, i, cluster.only = TRUE, diss = TRUE)

df <- data.frame(as.list(pam))

write.table(df, file = paste('pam.', i,'.clusters.list', sep = ''))

# Calculation of the Silhouette coefficient

sil <- silhouette(pam, dist = d.dist)

print(paste('************', i, 'clusters *****************************', sep = ' '))

print(summary(sil))

}

Output:

[1] "************ 2 clusters *****************************"

Silhouette of 283 units in 2 clusters from silhouette.default(x = pam, dist = d.dist) :

Cluster sizes and average silhouette widths:

44 239

0.1262367 0.2824475

Individual silhouette widths:

Min. 1st Qu. Median Mean 3rd Qu. Max.

-0.09957 0.19816 0.28971 0.25816 0.34442 0.40069

[1] "************ 3 clusters *****************************"

Silhouette of 283 units in 3 clusters from silhouette.default(x = pam, dist = d.dist) :

Cluster sizes and average silhouette widths:

42 227 14

0.0732042 0.2831696 0.4476534

Individual silhouette widths:

Min. 1st Qu. Median Mean 3rd Qu. Max.

-0.2197 0.2023 0.2896 0.2601 0.3424 0.6132

[1] "************ 4 clusters *****************************"

Silhouette of 283 units in 4 clusters from silhouette.default(x = pam, dist = d.dist) :

Cluster sizes and average silhouette widths:

34 206 15 28

0.1176492 0.0889015 0.4208414 0.1788044

Individual silhouette widths:

Min. 1st Qu. Median Mean 3rd Qu. Max.

-0.09507 0.05809 0.10803 0.11884 0.13898 0.59392

[1] "************ 5 clusters *****************************"

Silhouette of 283 units in 5 clusters from silhouette.default(x = pam, dist = d.dist) :

Cluster sizes and average silhouette widths:

32 122 86 15 28

0.12374769 0.02117920 0.02083379 0.41852543 0.16924196

Individual silhouette widths:

Min. 1st Qu. Median Mean 3rd Qu. Max.

-0.07921 0.01147 0.03038 0.06838 0.06193 0.59300

[1] "************ 6 clusters *****************************"

Silhouette of 283 units in 6 clusters from silhouette.default(x = pam, dist = d.dist) :

Cluster sizes and average silhouette widths:

31 119 83 9 14 27

-0.02303518 0.02293401 0.01884178 0.50997309 0.44354584 0.18114083

Individual silhouette widths:

Min. 1st Qu. Median Mean 3rd Qu. Max.

-0.355559 0.007088 0.028612 0.068089 0.060055 0.644909

[1] "************ 7 clusters *****************************"

Silhouette of 283 units in 7 clusters from silhouette.default(x = pam, dist = d.dist) :

Cluster sizes and average silhouette widths:

22 108 74 30 9 13 27

0.11872367 0.02721710 0.01085489 -0.02535674 0.50737767 0.50056818 0.17348201

Individual silhouette widths:

Min. 1st Qu. Median Mean 3rd Qu. Max.

-0.356975 0.007864 0.031533 0.075448 0.072800 0.651127

[1] "************ 8 clusters *****************************"

Silhouette of 283 units in 8 clusters from silhouette.default(x = pam, dist = d.dist) :

Cluster sizes and average silhouette widths:

22 44 78 62 28 9

13 27

0.0968624138 -0.0001062449 0.0543973922 -0.0009122827 -0.0213879889 0.5051710954

0.4988263583 0.1555986775

Individual silhouette widths:

Min. 1st Qu. Median Mean 3rd Qu. Max.

-0.346712 -0.001711 0.043497 0.074015 0.092088 0.649610

[1] "************ 9 clusters *****************************"

Silhouette of 283 units in 9 clusters from silhouette.default(x = pam, dist = d.dist) :

Cluster sizes and average silhouette widths:

22 42 76 55 24 9 13

27 15

0.083829421 -0.003613566 0.038253838 0.021455973 -0.019270606 0.501369352 0.495881160

0.148204162 0.017224283

Individual silhouette widths:

Min. 1st Qu. Median Mean 3rd Qu. Max.

-0.326040 0.005828 0.037801 0.072565 0.084375 0.647821

[1] "************ 10 clusters *****************************"

Silhouette of 283 units in 10 clusters from silhouette.default(x = pam, dist = d.dist) :

Cluster sizes and average silhouette widths:

22 38 68 45 24 24 9

13 25 15

0.07563199 -0.00529747 0.04093448 0.02145577 -0.01997563 0.02685424 0.50136935

0.49550631 0.15114960 0.01257174

Individual silhouette widths:

Min. 1st Qu. Median Mean 3rd Qu. Max.

-0.326040 0.003932 0.037419 0.071724 0.083878 0.647821

**Machine learning**

In this study, we used three machine learning approaches. As predictive models, we used Predictive Clustering Trees (PCTs) for hierarchical multi-label classification (Blockeel et al.; Vens et al., 2008) and Random Forest ensembles thereof (Kocev et al., 2013)**.** To evaluate the importance of individual features, i.e., input attributes, we employed a feature ranking method, which first constructs a Random Forest ensemble of PCTs and then uses the Genie3 (Huynh-Thu et al., 2010) scoring to calculate contributions of individual input features towards learning the output features. We use a feature ranking method specific to the task of hierarchical multi-label classification. We describe all three methods below.

**Predictive Clustering Trees (PCTs).**

PCTs are generalized decision trees, implemented in the CLUS software (Blockeel and Struyf), where a tree is viewed as a hierarchy of clusters (nodes). The root node represents one large cluster of all data which is recursively split into smaller clusters. The tree is partitioned by calculating the heuristic value of every possible test (split) and selecting the one with the best heuristic value. The partitioning continues until a stopping criterion is met. Nodes (clusters) at the bottom are called leaf nodes and provide predictions. The heuristic that guides the test (split) selection aims to minimize the intra-cluster variance of the target variables averaged over the subsets induced by the test. Lower average variance in the resulting clusters yields more accurate models.

PCTs can address several machine learning tasks. In this paper, we use the PCT learning algorithm for solving the **hierarchical** **multi-label classification** (HMLC) task, where the target (output) features are binary labels (which can be present or not) that are organized in a predefined hierarchy. The label hierarchy for our task is shown in Fig. 1. The learning examples (subjects in our case) can be annotated with more than one label, e.g., hospitalized (yes/no), tumor (yes/no), infection (yes/no), IBD (yes/no), Chron’s disease (yes/no), ulcerative colitis (yes/no), other (yes/no). In HMLC, labels are hierarchically taxonomically ordered and consequently not all label combinations are valid: If an example has a given label *L*, it also needs to have all the parent labels of *L* from the taxonomy. We exploit the label hierarchy during model training, considering similarities between subjects in terms of labels at higher levels of the hierarchy more important (by a weight of *w*) than similarities in terms of labels at lower levels of the hierarchy. We impose the so-called hierarchy constraint also when making predictions: If the model predicts a given label of the hierarchy, it also predicts all of its parent labels, e.g., if a PCT predicts the label ‘CD’, it will also predict ‘IBD’, ‘HPs’ and the meta-label *Subject*. The meta-label *Subject* is always predicted for all examples. Leaf nodes of the PCTs predict parts of the aforementioned hierarchy. It is important to note that the label hierarchy is separate from (and should not be confused with) the hierarchical structure of PCTs.

Supplementary Figure 6 shows the predictive clustering tree (PCT) induction algorithm. The overall induction process is described in Algorithm 1, to which we provide the data (E) as input. The tree is constructed by searching for the best possible split (Algorithm 2) in the currently considered data (line 1, Algorithm 1). If such a split is found, the data is divided according to it and the procedure repeats in a recursive manner (line 4, Algorithm 1) until no further splits can be discovered. Each discovered split becomes a test (node) in the decision tree, e.g., “*Age > 56”*. The tests are selected by evaluating all input attributes and all possible split points on them. Ultimately, the selected test is the one that reduces the impurity (*IM*) the most. The impurity reduction is calculated by subtracting the weighted sum of the impurity of the child nodes from the impurity of the parent node (line 4, Algorithm 2). The result of the induction (Algorithm 1) is a predictive model called a Predictive Clustering Tree. An example PCT model is shown in Supplementary Figure 7.


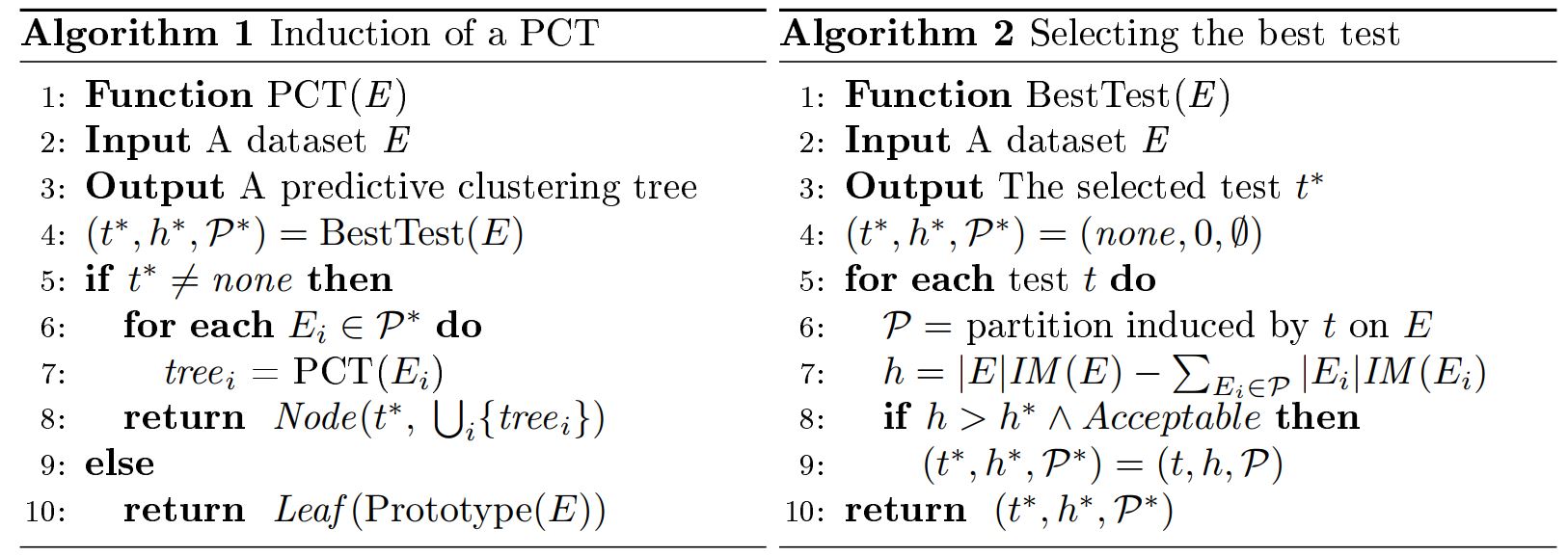


**Supplementary Figure 6:** The algorithm for constructing Predictive Clustering Trees (PCTs). Left: main algorithm, that recursively constructs the tree. Right: algorithm for selecting the best test, i.e., the best way to split the data. This procedure is called at every tree node.

**The Random Forest ensemble method**

The Random Forest ensemble method was used to lift the predictive performance of single PCT models by learning a set of PCT models. To this end, it uses bootstrap replicates of the training data (bootstrapping) to introduce randomization in the learning datasets for the individual ensemble models. It also uses a randomized version of Algorithm 2, which randomly selects input attribute subsets at each call of the BestTest procedure, introducing further randomization. This process of randomization is applied during the learning of all PCTs in the ensemble. The ensemble makes predictions with majority voting. Each PCT in the ensemble provides predictions for the labels of a given example (subject). The predictions are then averaged across the whole ensemble, yielding the final prediction for the given example (subject).

**Supplementary Figure 7:** An example Predictive Clustering Tree (PCT) for hierarchical multi-label classification. If a subject does not use antibiotics, it predicts the label ‘NHCs’. If the subject does use antibiotics and is more than 56 years old, it predicts the labels ‘HPs’ and ‘Other’. Finally, if the subject uses antibiotics and is 56 years old or younger, it predicts the labels ‘HP’, ‘IBD’ and ‘UC’. The label Subject applies to all subjects and is predicted in all cases.

**Feature importance**

Feature importance *values* were calculated as Genie3 scores (Huynh-Thu et al., 2010) from Random Forest ensembles of PCTs for HMC (Petković et al., 2017). As mentioned above, the reduction of variance is an indication of node split quality. According to Genie3, feature importance in a tree is proportionate to the variance reduction caused by the split on that feature: The larger the variance reduction, the better. Features that do not appear in the tree get zero scores. To calculate overall feature importances from a PCT ensemble for all input attributes (features), we first initialize the importance scores of all features to zero. We then traverse each PCT in the ensemble and use the Genie3 score to calculate the variance reduction score at every decision tree node, updating the importance scores of the features used in the tests at the respective nodes by adding the variance reduction scores to them. After repeating the calculation process for all PCTs in the ensemble, the features with highest scores represent the most important features. For better comparability between importance scores calculated from ensembles of different sizes, we divide all scores with the number of PCTs in the ensemble (100 in our case).

**Model evaluation**

We evaluate the quality of the trained models, i.e., estimate their performance on unseen subjects, with 10-fold cross validation. $AU\bar{PRC}$ (Area Under average Precision Recall Curve) was used as an overall measure of model quality across the entire hierarchy of target groups. In addition, $AUPRC$ values were calculated for each target group separately. The measure $AUPRC$ combines two important quality measures: precision and recall. It is more suitable for our purposes than the more commonly used Area Under the Receiver Operating Characteristic curve ($AUROC$), because it better addresses the imbalance in target group/class distribution.

**Parameters used for machine learning methods**

Predictive Clustering Trees (PCTs) were trained using all available input features and were allowed to grow until all leaf nodes contained only 1 example. The models were pruned using automatic F-test pruning to correct for overfitting. The p value was automatically selected from the possible values of 0.125,0.1,0.05,0.01,0.005 and 0.001. The hierarchical label weight was set to default ($w_{0}=0.75)$. Random Forest ensembles contained 100 PCTs, which were learned as described above, except that they were not pruned. Individual models (PCTs) in the ensemble were allowed to consider $\sqrt{D}$ input attributes (selected at random at every node), where D represents the number of all input features (attributes). The same Random Forest ensemble was used in combination with the Genie3 scorer to estimate the importance of input.

**References**

Blockeel, H., and Struyf, J. Efﬁcient Algorithms for Decision Tree Cross-validation. J. Mach. Learn. 2002;3:621-650.

Blockeel, H., Raedt, L.D., and Ramon, J. (1988). Top-down induction of clustering trees. Mach. Learn. 15, 55-63

Huynh-Thu, V.A., Irrthum, A., Wehenkel, L., and Geurts, P. (2010). Inferring regulatory networks from expression data using tree-based methods. PLoS One 5, e12776.

Kocev, D., Vens, C., Struyf, J., and Džeroski, S. (2013). Tree ensembles for predicting structured outputs. Pattern Recognition 46, 817–833.

Petković, M., Džeroski, S., and Kocev, D. (2017). Feature ranking for multi-target regression with tree ensemble methods. Discovery Science 171–185.

Vens, C., Struyf, J., Schietgat, L., Džeroski, S., and Blockeel, H. (2008). Decision trees for hierarchical multi-label classification. Mach. Learn. 73, 185.
